# Multi-intervention integrated deworming strategy for sustained control of soil-transmitted helminths infections: a case study in Jiangsu Province, China

**DOI:** 10.1186/s40249-021-00903-7

**Published:** 2021-09-10

**Authors:** Fan-Zhen Mao, Yu-Ying Chen, Xiang-Zhen Xu, Bi-Xian Ni, Xiao-Lin Jin, Yang Dai, Jun Cao

**Affiliations:** 1 National Health Commission (NHC) Key Laboratory of Parasitic Disease Control and Prevention, Jiangsu Provincial Key Laboratory On Parasite and Vector Control Technology, Jiangsu Institute of Parasitic Diseases, Wuxi City, Jiangsu Province 214064 People’s Republic of China; 2 Center for Global Health, Nanjing Medical University, Nanjing City, Jiangsu Province 211166 People’s Republic of China; 3 Public Health Research Center, Jiangnan University, Wuxi City, Jiangsu Province 214064 People’s Republic of China

**Keywords:** Soil-transmitted helminths, Multi-intervention, Control, Strategy, China

## Abstract

**Background:**

Soil-transmitted helminths (STH) infections still present a global health problem. Mass drug administration (MDA) is a widely applied strategy to reduce morbidity and mortality caused by STH. Yet, this approach has some shortcomings. In this study, we analyzed the impact of a multi-intervention integrated deworming approach including MDA, health education (HE), and environmental sanitation improvements (ESI) for sustained STH control in Jiangsu Province of China that was applied from 1989 to 2019.

**Methods:**

Data, including infection rate of STH, medications used, coverage of the medication, non-hazardous lavatory rate, and household piped-water access rate in rural areas, and actions related to HE and ESI were collected (from archives) and analyzed in this retrospective descriptive study. Pearson’s correlation analysis was applied to test correlations.

**Results:**

There was a dramatic decline in the infection rate of STH from 1989 (59.32%) to 2019 (0.12%). From 1995 to 1999, MDA and HE were recommended in rural areas. A negative correlation was observed between infection rate and medication from 1994 to 1998 (r = - 0.882, *P* = 0.048). From 2000 to 2005, targeted MDA was given to high-risk populations with HE continuously promoting good sanitation behaviors. From 2006 to 2014, targeted MDA + HE and ESI were used to consolidate the control effect. ESI was strengthened from 2006, and a negative correlation was observed between the coverage rate of the non-hazardous lavatory and the infection rate from 2006 to 2019 (r = - 0.95, *P* < 0.001). The targeted MDA was interrupted in 2015, while continuous efforts like HE and ESI contributed in sustaining STH control.

**Conclusions:**

Multi-intervention integrated deworming strategy contributes to the reduction of STH infections. This approach is a valuable example of how different interventions can be integrated to promote durable STH control.

**Graphic abstract:**

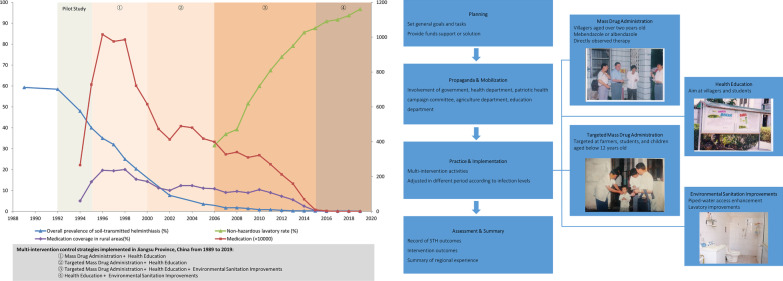

**Supplementary Information:**

The online version contains supplementary material available at 10.1186/s40249-021-00903-7.

## Background

Soil-transmitted helminths (STH) infection is a neglected tropical disease (NTD), largely affecting the rural areas in developing countries [1, 2]. The most common causative agents causing STH infection are the roundworm (*Ascaris lumbricoides*), whipworm (*Trichuris trichiura*), and hookworm species (*Ancylostoma duodenale* and *Necator americanus*). Community-based and/or targeted mass drug administration (MDA) of anthelminthic chemotherapy is widely used to reduce morbidity and mortality caused by STH. Evidence suggests that MDA can reduce morbidity by decreasing the prevalence and intensity of infection and limiting transmission within endemic communities [3–5]. MDA remains integral in controlling programs against STH due to its low cost and widespread availability [6, 7].

Yet, MDA also has some limitations. For example, MDA can only temporarily reduce the transmission of STH infection and cannot prevent reinfection without integrated control approaches, particularly for *A. lumbricoides* and *T. trichiura* [8]. As a result, MDA is reused in case of reinfection. However, repeated implementation of MDA may lead to the threatening of anthelmintics resistance [5, 9, 10]. Thus, MDA is highly beneficial in environments with low transmission risk and strong health systems. On the other hand, areas with high transmission risk settings may require high-coverage, high-frequency, and broader community-based treatment, with additional health education (HE) and environmental sanitation improvements (ESI).

Given the above shortcomings and potential risks, more and more researchers support recommendations for integrated control approaches. Combined with MDA, water, sanitation, and hygiene (WASH) interventions like HE and ESI can achieve durable control of STH [8, 11–14]. Multi-intervention integrated strategies, including MDA and WASH interventions, have been suggested by the World Health Organization to control STH [15], i.e., reduce STH transmission and reinfection. However, so far, only a few studies have reported on the feasibility of this approach. Moreover, whether these interventions should be applied in all settings, regardless of their epidemic levels, or whether they should be adjusted according to the specific circumstance of different settings, remains unclear. Multi-interventions need to be carefully planned and implemented as an integrated strategy; yet, this process is complex and expensive.

The prevalence of soil-transmitted helminthiases in China has caused large economic losses, especially in the 1990s when Jiangsu Province was considered the region with the highest prevalence of STH infections (in 1989, the first national prevalence survey of parasites reported a prevalence of 59.32%) [16]. Over the years, combined interventions, including MDA, HE, and ESI, have been performed in the Jiangsu area, drastically decreasing the rate of STH infection. The present study analyzed the impact of a multi-intervention integrated deworming strategy for sustained STH control in Jiangsu Province of China over the past 30-year period to provide a pragmatic and prospective viewpoint on deworming.

## Methods

### Study area

This is a retrospective descriptive study of a multi-intervention integrated deworming strategy for the STH control program implemented in Jiangsu Province, China, between 1989 and 2019. Jiangsu Province is located in the lower reaches of the Yangtze River, East of China, where temperature and humidity are favorable for the transmission of intestinal parasites. Farmers and rural villagers make up a large proportion of the population in this region; there were 84.2% farmers and rural villagers in 1982, 78.4% in 1990, and only 32.3% in 2016, respectively [17, 18]. STH control was conducted primarily in rural villages and towns with poor housing and sanitation, and a high-risk rate of STH transmission.

### Data collection

Data that were derived from archives were collected from 1989 to 2019. STH outcomes, including infection rate before and after treatment (in the pilot study), the annual infection rate of STH, medications used, and medication coverage in rural areas, were obtained from routine parasitological surveys conducted by municipal Centers for Disease Control and Prevention (CDC). A county or town was chosen in every municipal area. Each county or town selected a convenience sample of two villages with different infection (high and low) levels. Data were collected from at least 500 individuals per village. In each village, stool samples from all the eligible individuals (above two years old) were collected; samples were analyzed one month before MDA. In each village, there was at least one health bureau staff member responsible for parasitic diseases control. All staff members received training (education regarding the biology of STH and microscopy detection) at the Jiangsu Institute of Parasitic Diseases (JIPD) every year.

Sample detection was performed at the county or prefecture CDC laboratories. Fecal egg detection was performed using Kato-Katz thick smear technique (triple slide) as recommended by WHO [19]. An expert microbiologist reviewed all positive slides and 10% of negative slides at the JIPD; discrepant results were resolved by inviting a senior microbiologist from JIPD. All the data were recorded and summarized annually. STH outcomes were extracted and checked year by year from the historical archives by the staff from the County-level or City-level CDC according to the principle of authenticity and accuracy.

Data of ESI, which included annual non-hazardous lavatory rate and access rate to piped-water in rural areas, were acquired from Jiangsu statistic yearbook and Jiangsu Patriotic Health Campaign Committee Office.

### Main interventions

**Fig. 1:**
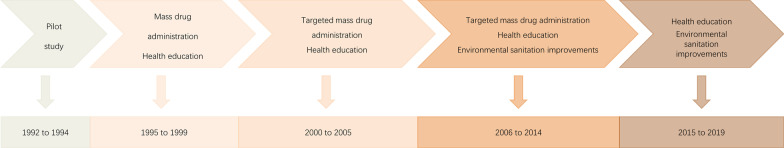
Timeline of the multi-intervention control strategies implemented in Jiangsu Province, China, from 1989 to 2019

During the 30-year study period between 1989 and 2019, a timeline of the multi-intervention control strategies was established for the control of STH in Jiangsu Province, China (Fig. 1). In rural areas, a pilot study was conducted from 1992 to 1994, and MDA and HE were recommended from 1995 to 1999. From 2000 to 2005, targeted MDA was given to high-risk populations with HE continuously promoting good sanitation behaviors. From 2006 to 2014, targeted MDA + HE and ESI were used together to consolidate the control effect. The targeted MDA was interrupted in 2015, while continuous efforts like HE and ESI contributed in sustaining STH control.

#### Mass drug administration (MDA)

MDA was primarily implemented in rural villages and towns. Pregnant women, individuals with severe acute or chronic illness, fever, or an allergy to mebendazole or albendazole, and those < 2 years old were excluded from the study. Mebendazole or albendazole pills (400 or 500 mg) were used according to WHO Guidance [20, 21]. Health officers directly observed therapy and recorded doses accordingly. The frequency of medication depended on the infection rate of STH in each village: if the prevalence was 50% or higher, MDA was offered biannually; if the prevalence was 20%–50%, MDA was offered annually; if the prevalence was ≤ 20%, individuals with positive stool tests were offered medication once a year. Stool specimens were collected from all eligible village members and analyzed using a Kato-Katz method. All positive slides and 10% of negative slides were reviewed for confirmation by an expert microbiologist from JIPD at the provincial laboratory.

In the pilot study considering the time period between 1992 and 1994, baseline investigations were conducted in each village to estimate infection of STH. Stool specimens were collected and analyzed among the same population who underwent two years’ therapy. Following the pilot study, MDA was implemented in primarily rural villages and towns starting from 1995 in Jiangsu Province, with the same inclusion criteria and stratified approach of medication frequency. In each village, community health workers and local public health officers visited homes and encouraged eligible individuals to take mebendazole or albendazole pills.

To maintain quality control, medications were obtained by JIPD from national pharmaceutical distributors. Well-trained health bureau workers systematically monitored the side effects.

#### Environmental sanitation improvements (ESI)

In accordance with a national “toilet revolution” campaign, which started in 2006, the government reformed the environmental sanitation systems. ESI related to deworming included providing household piped-water access and construction of household lavatories, all of which could block intestinal infectious disease transmission, including STH infection. Moreover, household latrines using three-cell septic tanks were also provided. They were co-financed by the local government and household owners [22, 23]. Parasite eggs were filtered, while fecal-urinary mixture precipitates and layers underwent anaerobic fermentation.

Rural development and socio-economic initiatives, lavatory improvements, and piped-water access were critical in breaking STH transmission cycles. ESI was inter-sectoral cooperation led by the government, health department, patriotic health campaign committee, department of agriculture, and educational department. Governmental and non-governmental organizations coordinated a lavatory improvement project and assessed the lavatory infrastructures and hygienic, environmental, and social benefits. In addition to routine sanitation improvements, JIPD worked as a technical assistant on parasite detection and stool specimens identification.

#### Health education (HE)

Health education and promotion primarily targeted rural residents and school children (< 12 years old) through radio broadcasts, informational leaflets, and exhibition boards by local health bureau workers with help from village committees and school teachers. Radio broadcasts were performed every month in villages. Informational leaflets were distributed to villagers and school children two to four times per year. Exhibition boards were generally located in public places out of the village committee. The topic and content of health promotion materials were usually updated seasonally and included information regarding transmission, symptoms, and prevention (handwashing with soap before eating and after defecation, wearing shoes and gloves to protect from contaminated soil, and using treated night soil as fertilizer on vegetable gardens) [24–28]. Unfortunately, awareness rate, behavior improvement, and attitude change were not completely recorded.

### Statistical analyses

Medication adherence per person (if a person took medication two times a year, it was counted as two persons). The overall prevalence of soil-transmitted helminthiases was also analyzed. STH infection was considered if an egg of STH was detected in the stool specimen regardless of species. A non-hazardous lavatory rate and access rate to pipe water were applied to indicate ESI. The non-hazardous lavatory rate was used to analyze the household lavatory improvement and access to pipe water to indicate domestic water improvement in rural villages. Latrines in schools and villages for shared use were not included. The correlations between medication and the overall prevalence of soil-transmitted helminthiases, between the coverage rate of the non-hazardous lavatory and the overall prevalence of soil-transmitted helminthiases, were also analyzed. The temporal resolution of the analysis was the year.

Data were double entered into Microsoft Excel version 2007 (Microsoft Corporation; Redmond, WA, USA) and then imported into SPSS version 13.0 (SPSS, Inc.; Chicago, IL, USA) to conduct the analyses. Pearson’s correlation analysis was applied to test correlations. The trend lines were generated by Microsoft Excel 2007. All the maps were generated by QGIS 3.0 (the Mapserver Foundation; Wilmington, DE, USA). We were unable to report data on STH by each species because of data loss.

## Results

### The pilot study confirmed the reduction of STH infections after MDA

To determine the MDA treatment approach, a pilot study was conducted in 11 villages from each prefecture of Jiangsu Province, representing the overall conditions of each prefecture from 1992 to 1994. In 1992, 13,816 people aged over 2-year-old were involved, and the overall infection rate of STH was 58.47%; in 1994, 6,418 were involved, and an overall infection rate was 11.38%.

The side effect rate of the mebendazole and albendazole was 5.81%; the majority included mild gastrointestinal symptoms, dizziness, and headache. In most cases, the discomfort was alleviated within two days without any treatment. All the respondents showed enough understanding after careful observation and patient explanation. Moreover, villagers in pilot sites were likely to accept the chemotherapy against STH, especially children. Through 3 years’ pilot work, infection rates of STH significantly decreased (Table 1 shows the details of four pilot sites).

**Table 1:** Changes of soil-transmitted helminth infections (hookworms, roundworms, and whipworms) in Jiangsu pilot sites during 1992–1994

Year	Xinghua County of Yangzhou City		Jintan District of Changzhou City		Rudong County of Nantong City		Wuxian County of Suzhou City	
	Sample (positive)	Infection (95% *CI*)	Sample (positive)	Infection (95% *CI*)	Sample (Positive)	Infection (95% *CI*)	Sample (positive)	Infection (95% *CI*)
1992	1329 (709)	53.3 (50.7–56.0)	967 (262)	27.1 (24.3–29.9)	1131 (486)	43.0 (40.1–45.9)	899 (196)	21.8 (19.1–24.5)
1993	500 (19)	3.8 (2.1–5.5)	338 (38)	11.2 (7.9–14.6)	409 (84)	20.5 (16.6–24.5)	184 (8)	4.3 (1.4–7.3)
1994	511 (14)	2.7 (1.3–4.2)	310 (19)	6.1 (3.5–8.8)	–	–	–	–

### A decreasing trend of infections correlated with expanded MDA

**Fig. 2:**
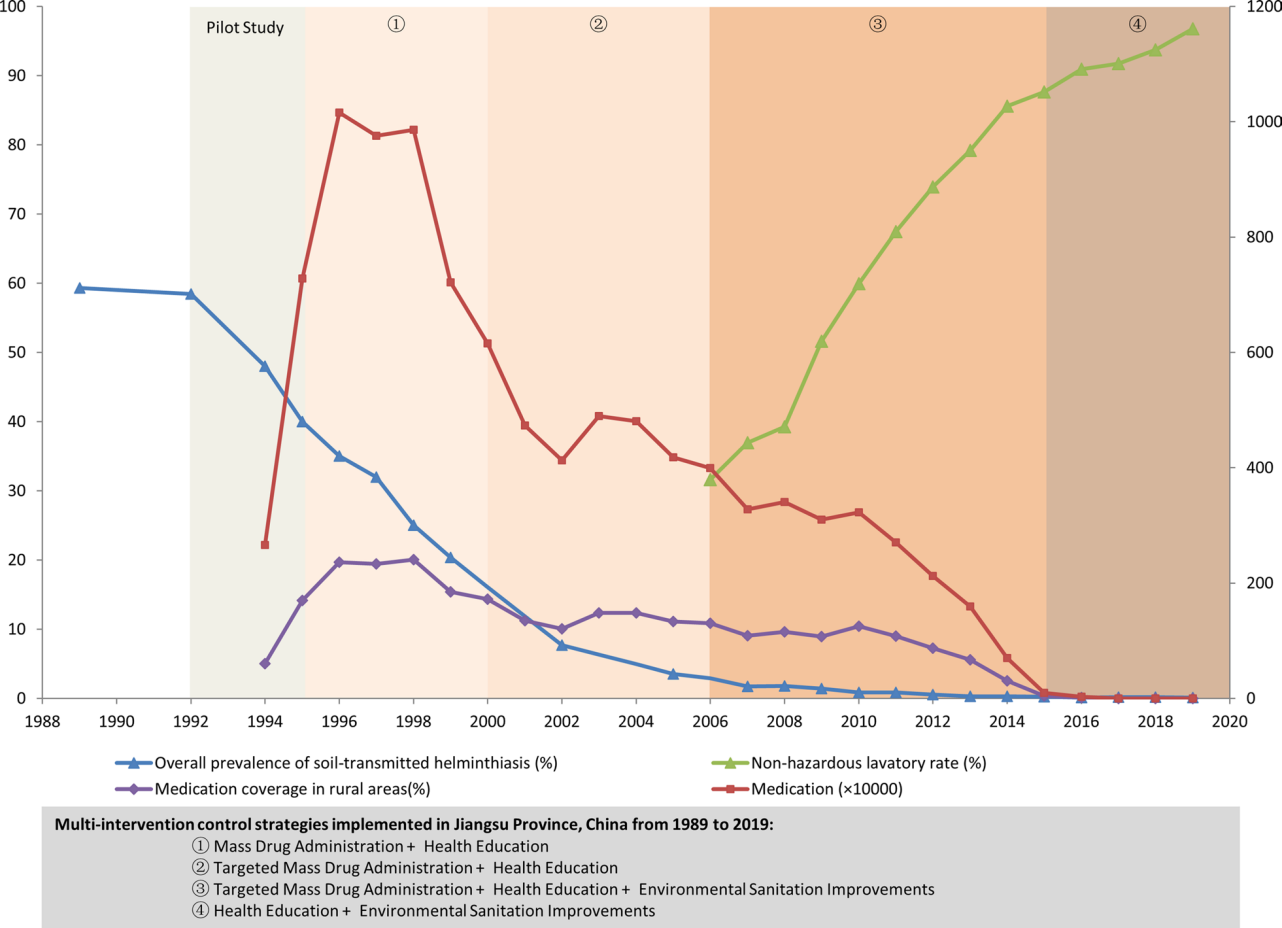
Multi-intervention integrated deworming strategy for sustainable control of soil-transmitted helminths in Jiangsu Province, China, from 1989 to 2019

From 1995 to 1999, the application of MDA and HE was conducted in rural areas of Jiangsu Province. Data showed a descending trend. In 1989, the overall infection rate was 59.32%, while in 2000, it was 16.10% (Fig. 2). Most medications were taken from 1996 to 1998; medicines were given to approximately 10 million people each year. Moreover, the infection rate and the medication showed a significantly negative correlation from 1994 to 1998 (r = - 0.882, *P* = 0.048). The coverage of medication in rural areas peaked in 1998, with a coverage rate of 20.05%. A conservative estimate of the total medications was 44.27 million people from 1995 to 2000.

### Targeted MDA consolidated the decreasing trend of infections

Considering the cost-effectiveness, due to the dramatic decline of soil-transmitted helminthiases prevalence from the year 2000 to 2005, the government decided to recommend the use of MDA (targeted MDA) only for high-risk populations, including those with positive stool tests, children (2–12 years old), and farmers who were at high risk of exposure to contaminative soil. From 2001 to 2005, the total amount of medications was 22.74 million people. HE was conducted during that time to help promote good sanitation habits. The infection rate of STH further declined to 2.92% in 2006 (Fig. 2).

### ESI blocked transmission and rebound in infection

In 2006, only 31.64% of households in villages were equipped with non-hazardous lavatories (Fig. 2), while in 2014, the number increased to 85.57%. The coverage rate of non-hazardous lavatory was negatively correlated with the infection rate from 2006 to 2019 (r = - 0.95, *P* < 0.001). The total amount of medications was 24.13 million people from 2006 to 2014. In 1995, the access rate to piped water in rural areas was 53.3%. Through incremental efforts, the rate increased to 80.2% and 95.2% in 2002 and 2005, respectively. Currently, more than 98% of people have access to piped water.

### Unceasing efforts contributed to maintaining a low level of infection rate

Targeted MDA was stopped in 2015 since the infection rate of STH was low (the infection rate was kept below 0.5% since 2013). However, constant efforts such as HE, surveillance, parasitology surveys of sentinel sites, and ESI were continued and especially emphasized following targeted MDA cessation.

With over 10 years of effort, there were 96.79% of households with non-hazardous lavatories in 2019. Today, more than 98% of people have access to piped water. The dramatic changes followed the rural environmental reform and stressing the importance of exterminating these poverty-related diseases that might impede public health.

### Regional differences of STH infection in Jiangsu Province

The decreasing pattern of STH infection in Jiangsu Province is clear during three different times (Additional file 2: Figure S1a–d). In 1989, prefectures in the north of Jiangsu Province initially showed a general high infection rate at the beginning of deworming and control, especially Xuzhou City, Lianyungang City, Suqian City Huai’an City (all above 70%). Zhenjiang City, Changzhou City, and Wuxi City, located in the south of Jiangsu, showed infections with 40–50% (49.3%, 43.4%, and 46.0%, respectively). In 2002, after implementing the targeted MDA, the infection rates of STH dramatically decreased (except for Suqian City, 22.6%), with a prevalence of < 20% (1.5–14.9%). Then, in 2015, the targeted MDA was stopped. Suqian City still ranked the first in the infection rate of STH (2.0%), with all other prefectures showing an STH infection rate of < 0.5% (0–0.6%). In 2019, all prefectures except Xuzhou (0.57%) in Jiangsu showed an STH rate of < 0.5% (0–0.47%) (details are shown in Additional File 1). Although regional differences exist in Jiangsu Province, the infection of STH showed a remarkable decline after implementing the multi-intervention integrated deworming strategy.

### Annual routine control schedule for STH in Jiangsu Province, China

**Fig. 3:**
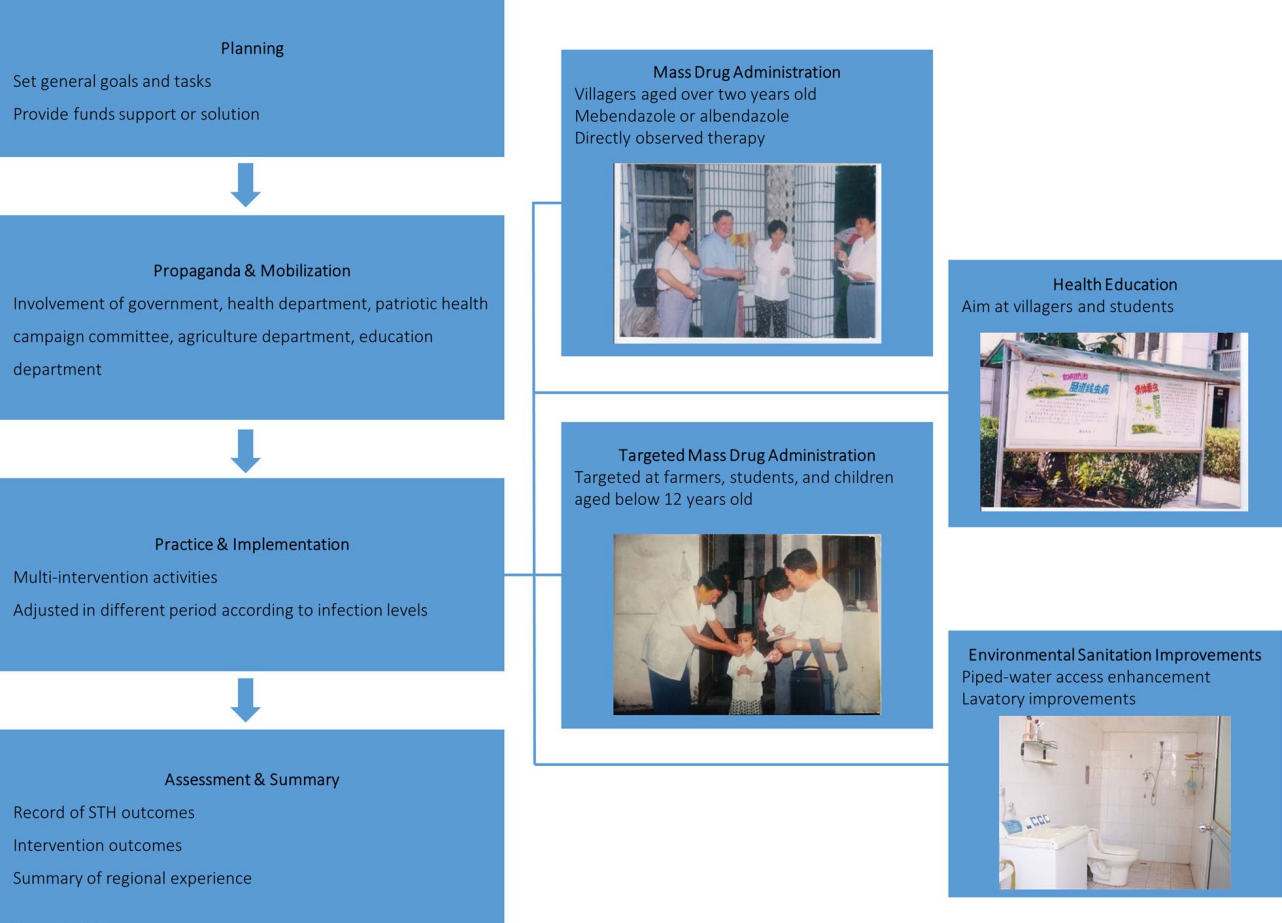
Annual routine schedule for STH control in Jiangsu Province, China. *STH* soil-transmitted helminth

Multi-intervention strategies have been undertaken throughout the province since 1989, including MDA, HE, ESI, monitoring, and assessment, with major measures adjusted for control in different infection levels (an overall infection rate of over 20%, between 20 and 5%, between 5% and 0.5%, and below 0.5%). Figure 3 shows the annual routine control schedule for STH in Jiangsu Province, China. The annual routine control schedule consisted of planning, propaganda and mobilization, practice and implementation, and finally, assessment and summary. By the end of 2019, a low infection level was achieved in all the counties in Jiangsu Province via annual routine efforts.

## Discussion

Following the implementation of 30-year multi-intervention strategies, including MDA, HE, ESI, and other involving actions, the infections of STH in Jiangsu Province, China, decreased from 59.32% in 1989 to 0.12% in 2019. Moreover, the infection rate has been stably maintained below 0.5% since 2013.

MDA is the main way to achieve rapid, substantial reductions in STH infection and intensity [29, 30]. Yet, MDA cannot prevent reinfection. Previous studies have suggested that school-based deworming (once a year) may not be adequate for controlling STH [31, 32]. Moreover, other studies suggested that MDA treatment might not be enough to reduce soil-transmitted helminthiases [3]. This effect could be reinforced by implementing ESI and HE, both of which are believed to block the cycles of STH transmission [11, 24, 25, 33–35] and consolidate the effect of MDA. Multi-intervention integration can be used to establish a degree of permanent infrastructure and sustainability and enhance utilization of existing resources. Such an approach also allows for flexible tailoring to community needs.

The parasitic infections are determined by several factors, i.e., environment, food habits, cultural tradition, social status, economic situation, and others. The prevalence of STH infection is a consequence of natural and cultural factors, but its control results from social consent and behavioral change. Thus, the combination and arrangement of the deworming interventions are likely to achieve a long-run effect without an unexpected rebound. In addition, a concomitant and effective surveillance-response system are still of utmost importance to effectively sustain the control and facilitate the progress towards the elimination of soil-transmitted helminthiases. Such a system identifies at-risk spots and underlying risk factors and prevents their reoccurrence, followed by readily tailored interventions to the specific social-ecological context.

Implementing STH control in a country demands social agreement to allocate appropriate resources, which must be supported by the government [12, 36]. With reference to STH infection in China, the Chinese government and health bureau established certain policies for STH control, placing great emphasis on deworming. Efforts were made by educational institutions, labor organizations, and the media to promote the deworming process. Piped-water supply and lavatory improvements were also reinforced and promoted by governments. Rather than solely being restricted to preventive chemotherapy targeting soil-transmitted helminthiases, comprehensive control requires inter-programmatic and inter-sectoral action for health and development [37–39]. However, many developing countries worldwide have limited resources, which is why the sustained prevalence of STH represents a substantial burden to developing countries [38].

STH infection is linked with low socioeconomic status, which presents a challenge for STH control. Information regarding the risk of STH infection is a key factor in preventing further transmission [40]. The overall success of decreasing STH infection is only partially attributable to MDA, as stated in studies conducted in Zanzibar and schistosomiasis MDA programs [41, 42]. Social and economic development are key drivers for controlling soil-transmitted helminthiases and other tropical parasitic diseases, for example, malaria. In Korea, nationwide biannual mass deworming targeting schoolchildren contributed to the dramatic decline of the STH prevalence rate from 84.3% in 1971 to a nadir of 2.4% in 1997 [38]. Nevertheless, economic growth, living standard improvement, and the implantation of sanitation and agricultural technology contributed to reducing STH infection. Currently, China is facing rapid socioeconomic development and large eco-environmental changes. The process of reform and opening up and the economy and extensive social efforts undoubtedly boosted the STH control effects, achieving a steady descent of STH infections without a rebound. The acceleration of industrialization and urbanization prompted the development of small towns and rural cities in China. Agricultural industrialization and improved living standards and sanitation consciousness gradually narrowed the gap between rural and urban areas in living habits and STH infections. Soil-transmitted helminthiases elimination program should be tailored to fit the socioeconomic development plan and the natural and environmental factors affecting STH transmission in the endemic regions.

Although there is a need for frequent MDA to maximize the benefit of preventive chemotherapy, integrated control approaches emphasizing HE and ESI are needed to interrupt STH transmission. In resource-limited settings, regular MDA is considered to be cost-effective [36, 43]. If resources allow, WASH interventions should be applied to promote behavioral change [44–46]. The current study presented a multi-intervention integrated deworming strategy for sustained STH control. According to different endemic levels, interventions were tailored to the social-ecological context. The evidence of deworming strategies from Jiangsu Province of China over 30 years have proved the feasibility of these approaches.

The general strategy requires inter-sectoral commitments from governmental and non-governmental organizations with strong public support. The public should be aware of the risks of STH infection and possible ways to control it. Public awareness could be improved by implementing HE and organizing systematic and continuous eradication programs. Experience from Jiangsu Province, China, has shown that mass chemotherapy with proper anthelmintics is essential. Anthelmintics should be inexpensive and convenient to use (dose, regimen, and frequency), especially when repeated treatments are required. Moreover, WASH interventions should be implemented to block STH transmission.

This study provides a new, different, and pragmatic perspective on how different interventions could be integrated into different stages of deworming, thus promoting a durative decrease of infection rate without a rebound. Moreover, STH control must be synergistic with a country’s development. As displayed, the medication coverage in rural areas was low even at its peak. Therefore, there is the possibility that a downward trend of STH infections can follow economic development as a result of improved sanitation and health consciousness, improved living conditions, and infrastructural improvements. This study showed no causative relationship between the prevalence of soil-transmitted helminthiases, MDA, and WASH interventions. Yet, the prevalence of soil-transmitted helminthiases should be associated with a multi-intervention integrated deworming strategy.

This study has a few limitations. Firstly, we did not report data on different STH species and the intensity of infections. Secondly, it is still unknown how much each intervention or action contributed in STH control, especially considering WASH, which includes household piped-water access and lavatory improvement in this study. Thirdly, the cost-effectiveness was not analyzed. Thus, it remains unclear whether these approaches are economical. Furthermore, although the annual routine control schedule could act as an “operation manual,” which was summarized according to Jiangsu’s practice and may be followed by some developing countries with high endemicity of soil-transmitted helminthiases, the cut-point about when to adjust interventions and how to adjust may not always be fixed in consideration of social context.

## Conclusions

Multi-intervention integrated deworming strategy contributed to the reduction of STH infections in Jiangsu Province of China. This approach is a valuable example of how different interventions could be integrated to promote durable STH control, especially in developing countries and endemic areas. The results of this study can be used to improve the design and realization of national deworming.

## Supplementary Information

**Additional file 1. **Data involved in this study.

**Additional file 2: Figure S1a–d.** Regional differences of the infection of soil-transmitted helminths in Jiangsu Province, 1989, 2002, 2015, and 2019. **a** Prefecture level prevalence in Jiangsu Province; the beginning of the deworming and control process, 1989. **b** Prefecture level prevalence in Jiangsu Province; implementing targeted mass drug administration and health education, 2002. **c** Prefecture level prevalence in Jiangsu Province; targeted mass drug administration stopped, 2015. **d** Prefecture level prevalence in Jiangsu Province; continuous interventions were conducted to achieve long-term control, 2019.

## Data Availability

All data generated or analyzed during this study are included in this article and its Additional information files.

## Abbreviations

STH: Soil-transmitted helminths
MDA: Mass drug administration
HE: Health education
ESI: Environmental sanitation improvements
