# Supplementary figures and images for: Multi-intervention integrated deworming strategy for sustained control of soil-transmitted helminths infections: a case study in Jiangsu Province, China
Source: Infect Dis Poverty. 2021 Sep 10;10:116. doi: 10.1186/s40249-021-00903-7 (PMC8434715; doi:10.1186/s40249-021-00903-7)

a


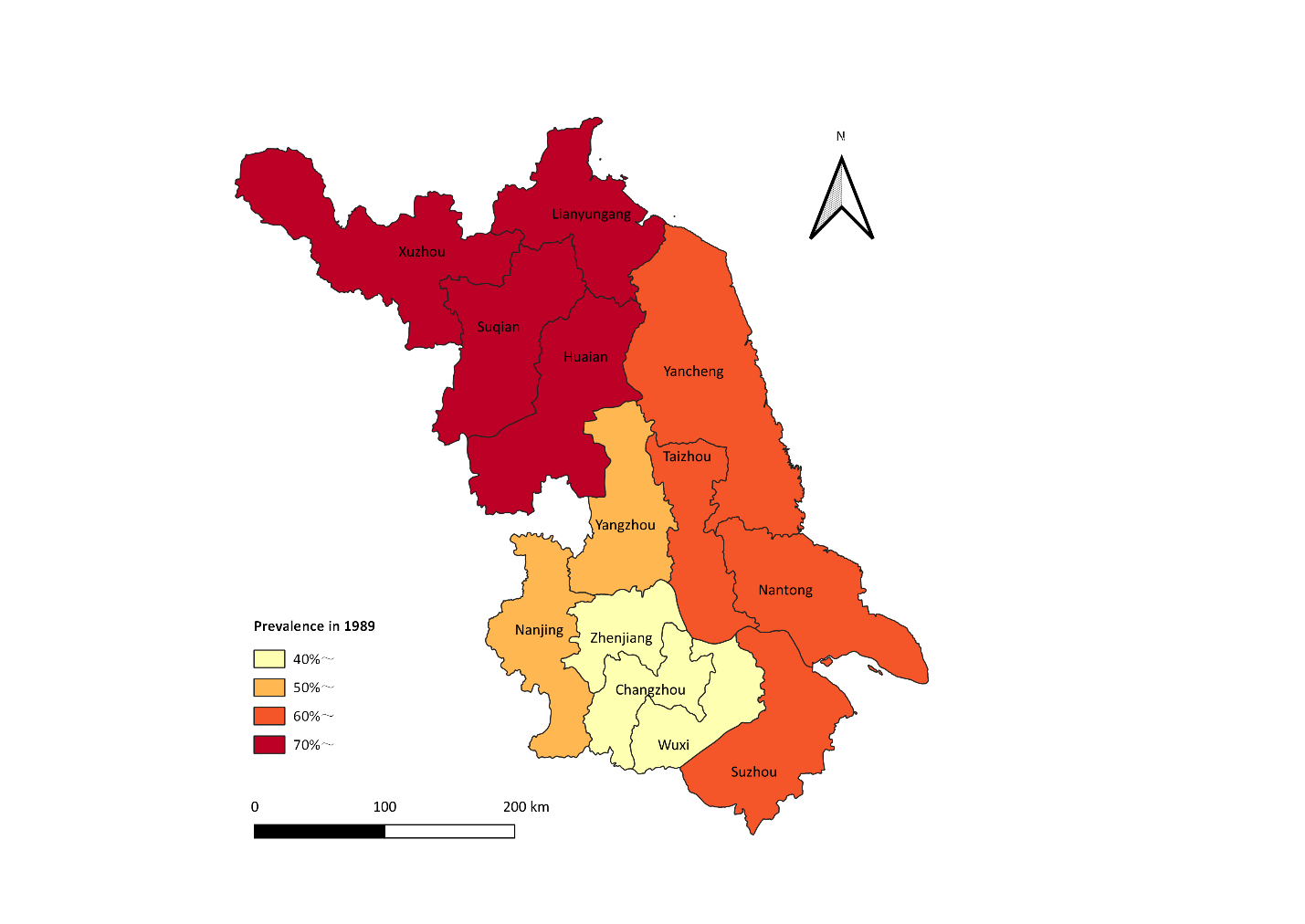


b


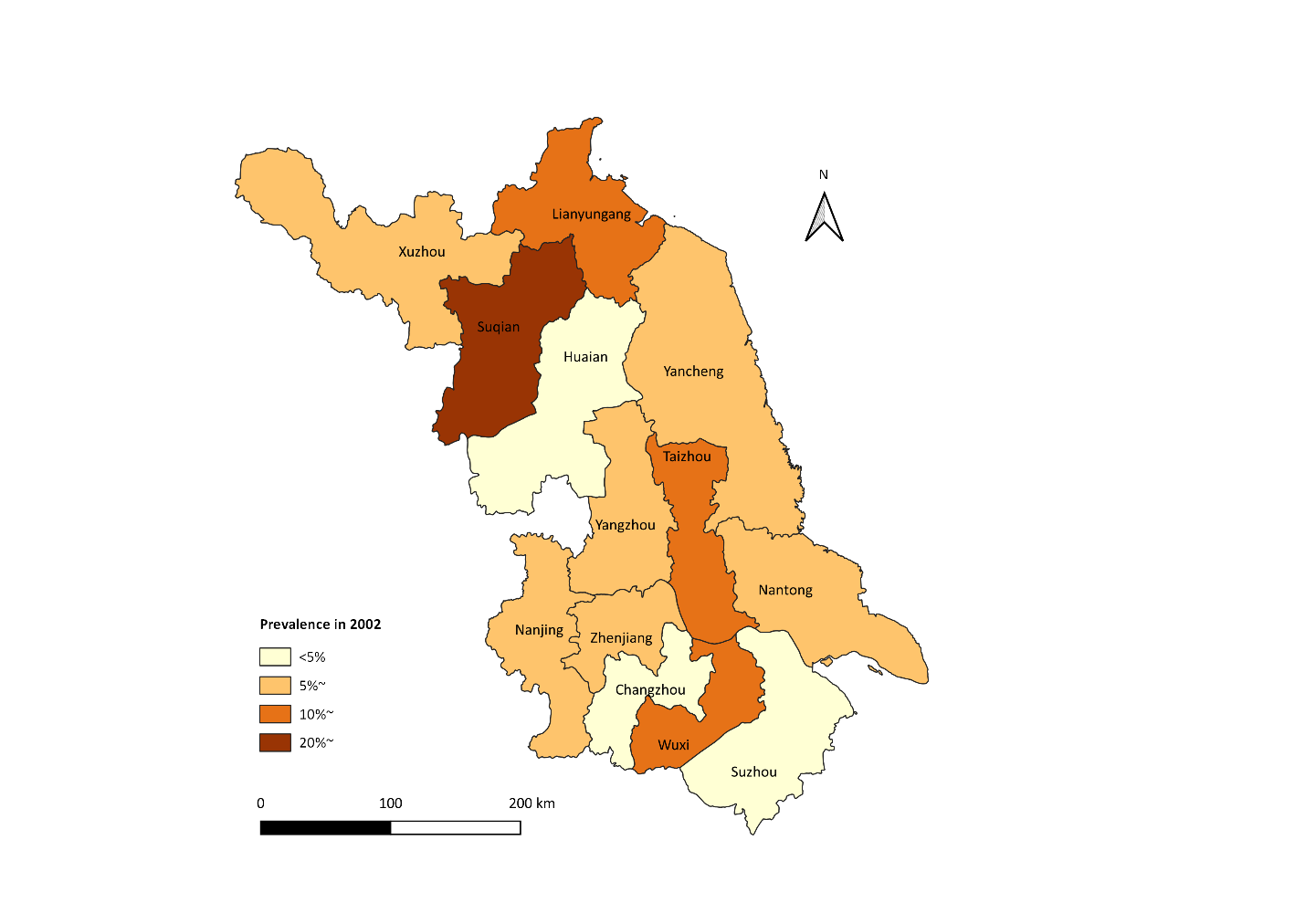


c


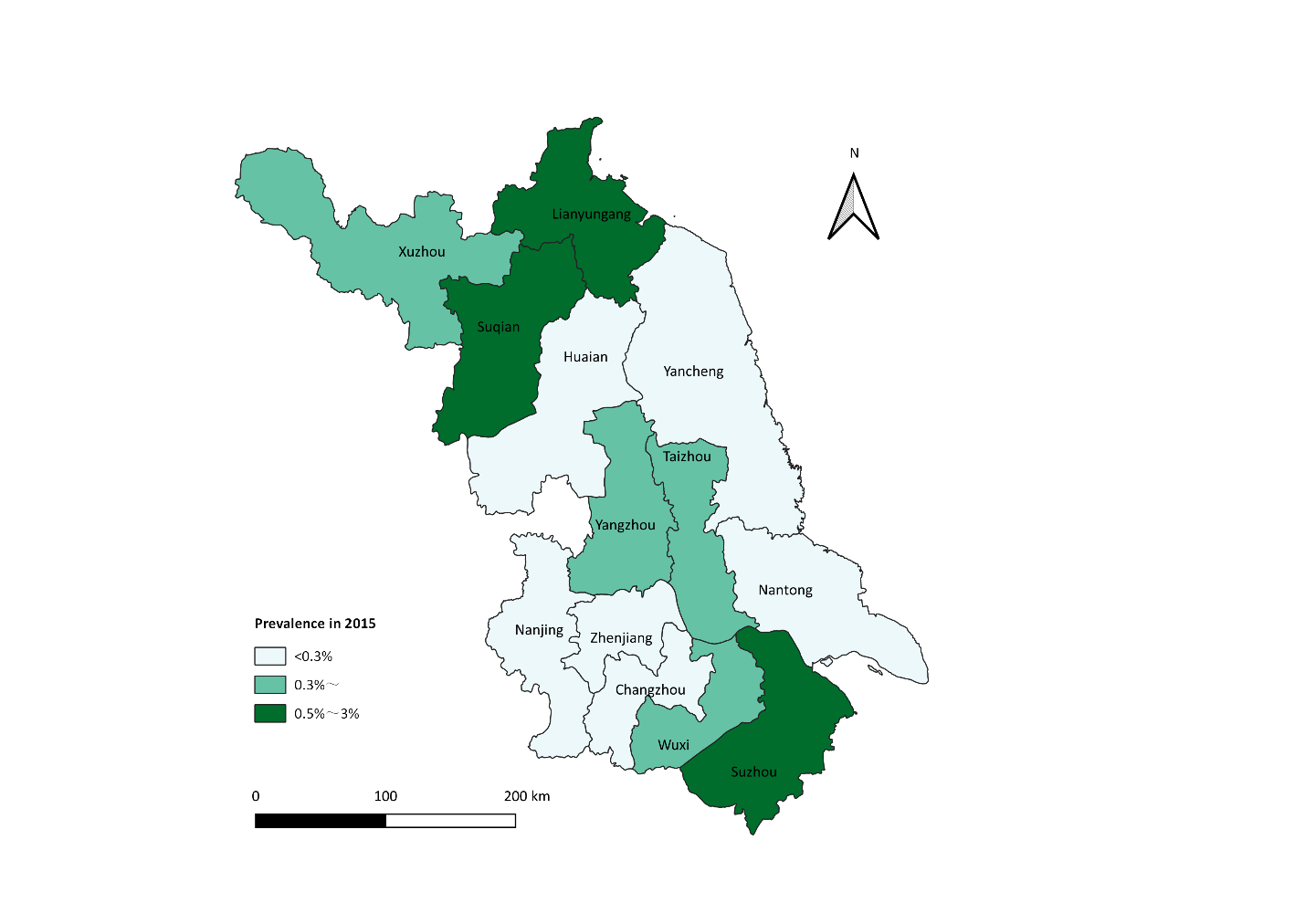


d


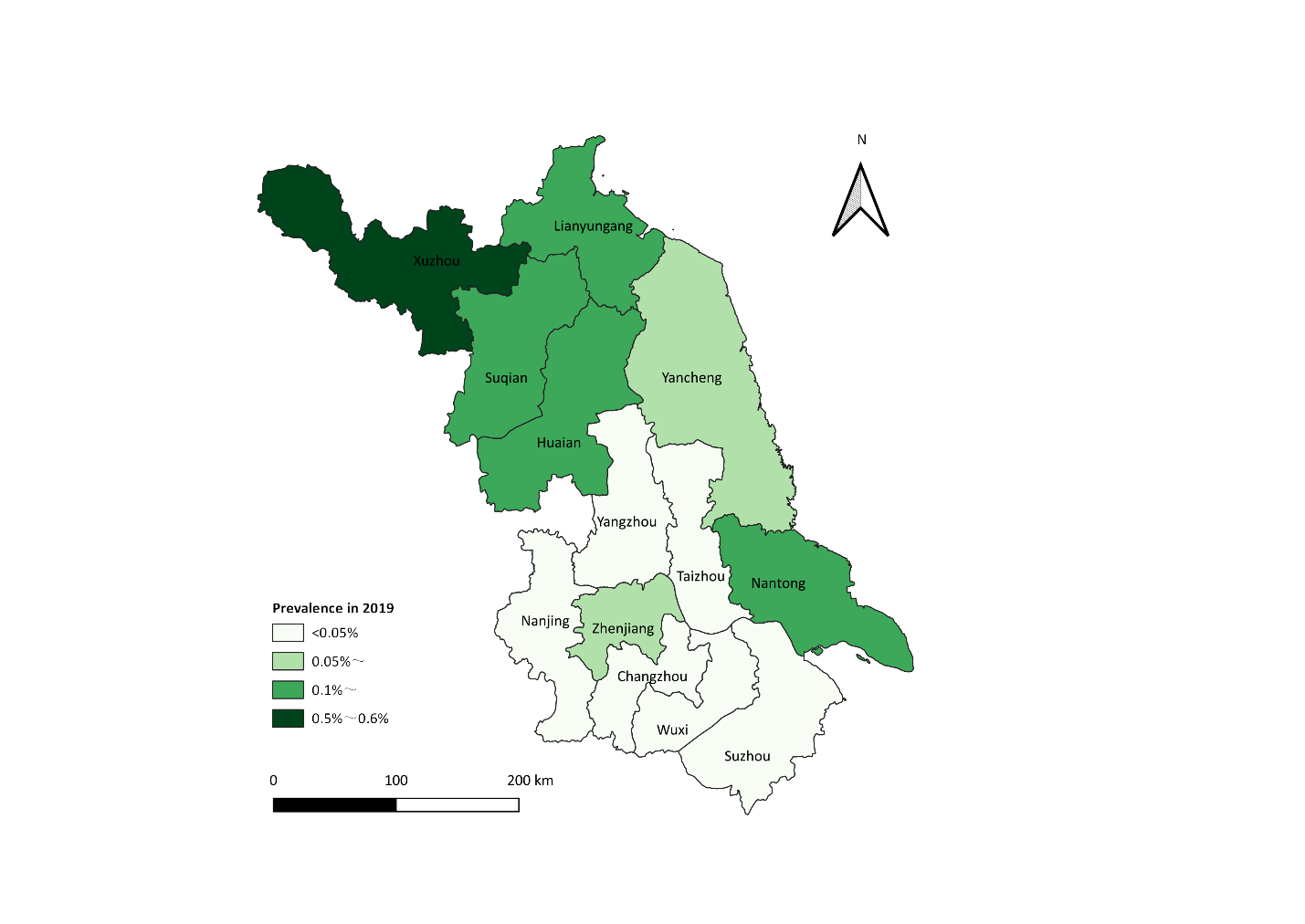

Supplement: Supplementary file 2 — Additional file 2: Figure S1a–d. Regional differences of the infection of soil-transmitted helminths in Jiangsu Province, 1989, 2002, 2015, and 2019. a Prefecture level prevalence in Jiangsu Province; the beginning of the deworming and control process, 1989. b Prefecture level prevalence in Jiangsu Province; implementing targeted mass drug administration and health education, 2002. c Prefecture level prevalence in Jiangsu Province; targeted mass drug administration stopped, 2015. d Prefecture level prevalence in Jiangsu Province; continuous interventions were conducted to achieve long-term control, 2019. [file 40249_2021_903_MOESM2_ESM.docx]
